# Supplementary material for: Social capital predicts corruption risk in towns
Source: R Soc Open Sci. 2019 Apr 3;6(4):182103. doi: 10.1098/rsos.182103 (PMC6502378; doi:10.1098/rsos.182103)
Supplement: Supplementary Information [file rsos182103supp1.pdf]

Supplementary information for: Social capital predicts corruption risk in towns

Johannes Wachs*[a], Taha Yasseri[b,c], Balázs Lengyel[d,e], and János Kertész[a,f]

[a]Department of Network and Data Science, Central European University, H-1051 Budapest, Hungary
[b]Oxford Internet Institute, University of Oxford, 1 St Giles, Oxford OX1 3JS, UK
[c]Alan Turing Institute, 96 Euston Road, London NW1 2DB, UK
[d]Agglomeration and Social Networks Lendület Research Group, Hungarian Academy of Sciences, H-1097 Budapest, Hungary
[e]International Business School Budapest, H-1037 Budapest, Hungary
[f]Institute of Physics, Budapest University of Technology and Economics, H-1111 Budapest, Hungary

March 7, 2019

1 Description of iWiW data

In line with previous work on iWiW we filtered the data used in our analysis. We use the data from the network at its peak activity in 2012. Out of roughly 4.5 million user accounts, we dropped the roughly 500,000 accounts with location outside of Hungary. We follow Lengyel et al. [6], we dropped the 193 users with more than 10,000 connections, arguing that such a large number of connections cannot represent social ties. We argue that this cutoff balances two concerns: it excludes those accounts with so many connections that it brings into question the nature of its connections, and we avoid truncating the tail of the distribution of social connectivity too much, allowing for sociality to range over several orders of magnitude. Many approaches to detect "fake" accounts in social network use the degree of a node as an important input [3].

In Plot A of Figure 1 we plot the sensitivity of fragmentation and diversity to the maximum degree threshold. If we discard all users with more than 100 connections (compared to the 10,000 connection cutoff we use in our paper), fragmentation would be significantly higher and diversity significantly lower than the versions we use in the paper. However this is not a reasonable cutoff as nearly 10% of users have more than 500 connections (see Plot B, Figure 1). The settlement fragmentation and diversity measures are within 5% of the versions we use in the paper if the threshold is set at 500, 1000, or 2000 connections.

In Figure 2 we show the relationship between settlement population and the number of iWiW users listing their location in the settlement, and the share of the population registered to iWiW.

As mentioned in the text, user privacy is a key concern. The anonymized iWiW data was made available to a consortium of researchers in Hungary, each of whom signed a non-disclosure agreement (NDA) to use the data for research purposes only. As a result, only settlement level aggregated data can be shared.

*Please direct all correspondence to johanneswachs@gmail.com

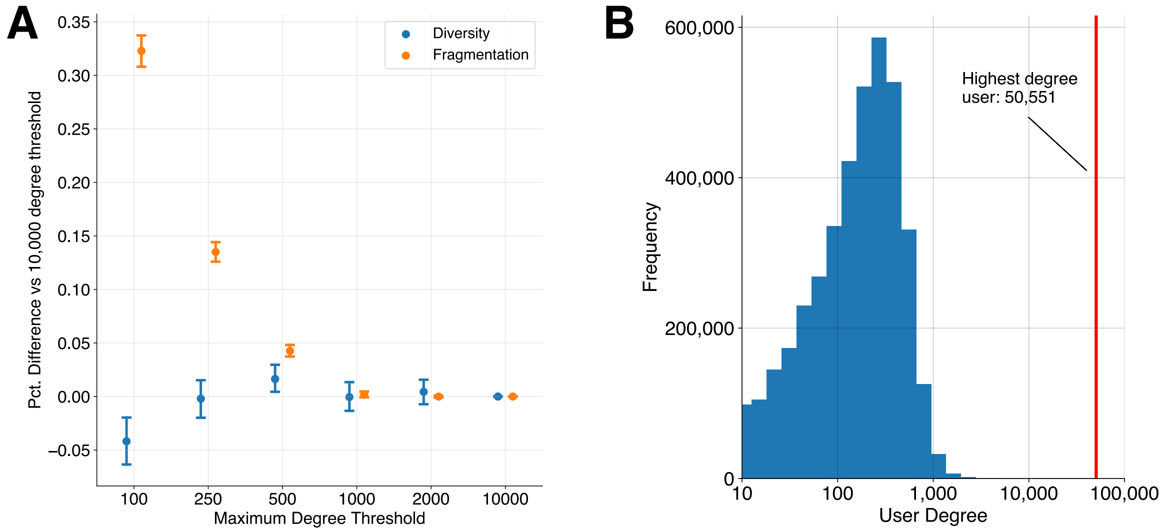

Figure 1: A) The sensitivity of diversity and fragmentation to changing the maximum degree threshold, relative to the 10,000 degree threshold used in the paper. Error bars represent 95% confidence intervals. The measures are within 5% of the version we use in the paper for cutoffs at or above 500. B) The distribution of user connections on a log scale. Very few users (193) have more than 10,000 connections, while many (405,337) have more than 500.

2 Corruption risk indicators

In this section we go into more detail regarding the individual corruption risk indicators. Each indicator quantifies different ways bureaucrats have excluded competitors in qualitative work on ground truth corruption cases from around the EU [4]. We stress that while no individual indicator or composite measure can credibly suggest that an individual contract was awarded by a corrupt process, aggregated over many contracts issued by the same institution these indicators map highly suggestive patterns. This point is an important motivation for filtering out towns awarding less than five contracts a year.

- Single bidder ($C_{singlebid}$) is an outcome: was the contract awarded in a competition attracting only a single offer.
- Closed procedure ($C_{closedproc}$ indicates when the contracting authority has decided to award a contract by direct negotiation with a firm or via an invitation-only bidding process. This decision can be used to completely subvert competition.
- No call for bids (C_{nocall}) indicates when, in the case that the contract was awarded via an open competition, no contract announcement or call for bids was published in the official procurement journal. A corrupt official can greatly decrease the chance of non-favored firms participating by limiting access to information.
- Long eligibility criteria ($C_{eligcrit}$) captures how bureaucrats can box out specific firms by adding requirements to participation criteria. By including many such restrictions (regarding previous experience, company size, qualifications), a corrupt bureaucrat can systematically exclude non-favored firms.
- Extreme decision period ($C_{decidetime}$ highlights suspicious activity between the end of a competition and the decision to award a contract. If the decision period is extremely short, this

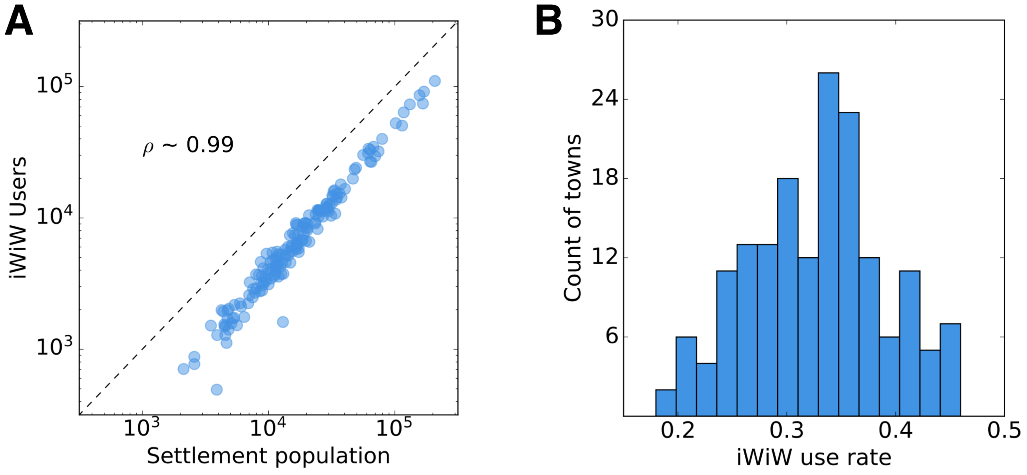

Figure 2: A) Settlement population and number of iWiW users plotted on a log-log scale. B) iWiW use rate by settlements.

suggests that the decision to award a specific firm was premeditated, and that the bids were not carefully checked. If the decision period is very long, it may indicate that legal challenges about the contract may be delaying the award decision.

- Short time to submit bids ($C_{bidtime}$) indicates that favored firms may have been tipped off about a competition for tenders ahead of the public announcement. By leaving only a short time between the announcement and the award for non-favored firms, the corrupt official makes it very difficult to submit a bid. It is important to remember that bids are complex legal documents, including at times cost estimates, schematics, and references.

- Non-price criteria ($C_{nonprice}$) tracks the share of non-price related or subjective criteria in the evaluation of bids. For instance, a corrupt bureaucrat may reject a lower cost bid if, according to a subjective criteria of the quality of a bid, it is less favorably evaluated than that of a higher cost bid of a favored firm.

- Call for bids modified ($C_{callmod}$) checks to see if a call for bids was modified between the initial announcment and the deadline. This potential corruption strategy closely emulates $C_{bidtime}$ in that a corrupt official can suddenly change the specifications or rules of a tender shortly before the deadline.

3 Relationship between fragmentation and diversity

Fragmentation and diversity, our measures of bonding and bridging social capital respectively, are positively and significantly correlated ($\rho \approx 0.46$). Though fragmentation considers only edges within the settlement and ego diversity includes external edges, both variables measure modularity in the network. However, according to our hypotheses, they are expected to capture different kinds of socialization. We found that despite their positive correlation these features have opposite relationships with our corruption risk measures: high fragmentation is positively and high diversity is negatively correlated with corruption risk. To test whether inter-settlement edges or the ego focus of diversity does more to distinguish the measure from fragmentation we recalculated the diversity considering

only edges within the settlement. This alternative "internal" diversity measure is weakly correlated ($\rho \approx 0.28$) with fragmentation, and strongly correlated with diversity ($\rho \approx 0.72$). This suggests that both the connections to other settlements and the ego-focus of the diversity measure distinguish fragmented settlements from diverse ones.

4 Model covariates and controls

In this appendix section we present the settlement-level variables used as controls in our models. We also report their summary statistics. Note that in our models, we scale all features to have mean 0 and standard deviation 1. Our controls mostly refer to data from 2011, when the last large scale Hungarian census took place and the data are of highest quality.

- *Average income per capita (2011)*: Wealthier places tend to be less corrupt [7] as competition for limited resources is expected to create greater incentive to cheat. Data on median income or the income distribution at the settlement level were, to the best our knowledge, not available in Hungary.
- *Population (log)(2011)*: Larger cities may have different contracting needs, different political and social norms, and different network characteristics.
- *Number of contracts awarded (log)*: Settlements contracting more frequently may be more experienced and may follow better practices. As more people are involved in contracting, corruption may become more difficult.
- *Rate of iWiW use (2012)*: The rate of iWiW use both proxies for the economic development of the settlement and controls for differences in observed social network structure resulting from differences in access to the web. Previous work suggests that iWiW users, especially the early adopters, skew young and wealthy [6].
- *Average mayoral victory margin*: Measured across three elections (2002, 2006, 2010), this variable proxies for the lack of political competition in the settlement. The absence of political competition has been shown to correlate with corruption [1].
- *Share of population with at least a high school diploma (2011)*: Education is typically correlated with better control of corruption [9].
- *Share of working-age population inactive and unemployment rate (2011)*: Counting the long-term and short-term unemployed respectively, these variables quantify economic stagnation. The economic hardship connected with high unemployment is conjectured to worsen political corruption [10].
- *The minimum travel distance to Budapest, the capital city*: This variable captures the physical isolation of the settlement from the main economic, political, and social hub of the country. Past research has shown that geographic isolation reduces accountability and increases corruption [2].
- *Share of population over 60 years old (2011)*: This variable controls for the over-representation of the elderly. The elderly are underrepresented on online social networks and tend to use these platforms differently than younger users [8].
- *Whether the settlement has a university (2011)*: This variable controls for the presence of a place of higher education in the settlement, including local branches of universities headquartered elsewhere. this which inflates the number of young people, hence likely iWiW users in the settlement.

Statistic	N	Mean	St. Dev.	Min	Max
Closed procedure or single bidder	169	0.59	0.15	0.21	0.92
Average CRI	169	0.28	0.04	0.16	0.40
Fragmentation	169	0.32	0.04	0.16	0.46
Avg. ego diversity	169	0.35	0.07	0.20	0.51
Income per capita (thousands HUF)	169	823.57	189.93	488.44	1,516.55
N contracts (log)	169	4.52	0.69	3.69	6.42
Population (log)	169	9.72	0.89	7.66	12.24
Rate iWiW use	169	0.33	0.06	0.18	0.46
Average mayoral victory margin	169	0.15	0.14	0.00	0.64
% high school graduates	169	47.23	10.22	25.70	76.80
Distance to Budapest (minutes)	169	114.00	54.34	22.55	228.57
Share of population inactive	169	0.30	0.04	0.20	0.40
Unemployment Rate	169	0.06	0.01	0.03	0.09
Share of population 60+	169	0.24	0.03	0.15	0.39
Has university	169	0.25	0.44	0	1

Table 1: Descriptive statistics of key settlement-level variables and controls.

5 Model results, diagnostics, and feature importances

We present the full model results in Table 2. Note that all variables are standardized with mean 0 and standard deviation 1. This aids interpretation, for example: a one standard deviation increase in the settlement's mayor's average margin of victory increases corruption risk by roughly one quarter of a standard deviation. We also present models including only one of the two network measures in Table 3. The effect and significance of both features is preserved when the other is excluded.

The estimated coefficients of the control variables and their levels of statistical significance offer additional insight into the phenomenon of corruption risk. Wealthier settlements are in general less corrupt, though the effect is not significant for CRI. Rate of iWiW use is not related with corruption risk and this does not change when we include the social capital features. The average mayoral victory margin is a highly significant positive predictor of corruption risk. One potential explanation is that mayors, who do not face significant competition do not fear being voted out of office if they are corrupt. Similarly settlements that are far from Budapest, which our models predict to be significantly more corrupt, may be insulated from investigation by the central authorities simply by being out of the spotlight.

One potential source of bias in the coefficient estimates of multiple regression models is collinearity among the predictors. We test for multi-collinearity for each predictor using a variance inflation factor (VIF) test, defined as the ratio of variance in the full model over the variance of the single-predictor model. We run this diagnostic for each predictor used in models (2) and (4) in the main text and report the results in Table 5. A popular rule of thumb is that VIF values under 10 denote acceptable levels of correlation between variables [5]. As it is near our limit, we reran our analyses without the "Share of population inactive" control variable, finding no substantive change in our results. The relevant model tables are available on request.

We show the relative variable importances of Model (6) (column 6 in Table **??**), the fully specific model predicting average CRI, using an Analysis of Variance F-test in Figure 3. We include only terms with a significant ANOVA F-test. Though other features have stronger predictive power, the social network features are more useful in predicting corruption risk than economic variables like unemployment, inactivity, and average income.

Dependent variable:	% Closed or single bid.		Average CRI	
	(1)	(2)	(3)	(4)
Fragmentation		**0.263*****		**0.207****
(Bonding social capital)		(0.097)		(0.092)
Diversity		**−0.553*****		**−0.551*****
(Bridging social capital)		(0.176)		(0.168)
Income/capita	−0.262	−0.277*	−0.075	−0.096
	(0.169)	(0.162)	(0.161)	(0.155)
N contracts (log)	−0.313*	−0.314*	−0.685***	−0.697***
	(0.171)	(0.165)	(0.162)	(0.158)
Population (log)	−0.180	0.020	0.118	0.335**
	(0.143)	(0.166)	(0.136)	(0.159)
Rate iWiW use	0.045	0.037	0.122	0.107
	(0.137)	(0.132)	(0.130)	(0.126)
Mayor victory margin	0.278***	0.255***	0.303***	0.281***
	(0.089)	(0.086)	(0.085)	(0.082)
% high school grads	0.166	0.374*	−0.176	0.040
	(0.190)	(0.199)	(0.181)	(0.190)
Distance to Budapest	−0.021	−0.198*	0.061	−0.112
	(0.104)	(0.112)	(0.099)	(0.107)
Share of pop. inactive	−0.797***	−0.805***	−0.716***	−0.754***
	(0.229)	(0.229)	(0.218)	(0.219)
Unemployment Rate	0.239**	0.262**	0.299***	0.320***
	(0.118)	(0.113)	(0.112)	(0.108)
% population 60+	0.501***	0.491***	0.500***	0.503***
	(0.163)	(0.158)	(0.155)	(0.151)
Has university	0.351	0.294	0.431**	0.352*
	(0.220)	(0.221)	(0.210)	(0.211)
Constant	1.245*	1.206*	2.779***	2.790***
	(0.725)	(0.702)	(0.689)	(0.671)
Observations	169	169	169	169
Adjusted R^2	0.163	**0.230**	0.183	**0.243**
F Statistic	3.967***	4.859***	4.419***	5.142***

Table 2: Settlement-level regression results predicting two corruption risk indicators. For both dependent variables, the first columns (1) and (3) correspond to the base model, predicting corruption risk using only control variables, and the second columns (2) and (4) show results, when the social network features are included. Note that all features are standardized with mean 0 and standard deviation 1. Significance thresholds: *p<0.1; **p<0.05; ***p<0.01.

Dependent variable:	% Closed or single bid.			
	(1)	(2)	(3)	(4)
Fragmentation			0.233**	0.263***
(Bonding social capital)			(0.099)	(0.097)
Diversity		−0.505***		−0.553***
(Bridging social capital)		(0.179)		(0.176)
Income/capita	−0.262	−0.295*	−0.243	−0.277*
	(0.169)	(0.166)	(0.167)	(0.162)
N contracts (log)	−0.313*	−0.359**	−0.269	−0.314*
	(0.171)	(0.168)	(0.169)	(0.165)
Population (log)	−0.180	0.083	−0.257*	0.020
	(0.143)	(0.168)	(0.144)	(0.166)
Rate iWiW use	0.045	0.009	0.073	0.037
	(0.137)	(0.134)	(0.135)	(0.132)
Mayor victory margin	0.278***	0.259***	0.276***	0.255***
	(0.089)	(0.087)	(0.088)	(0.086)
% high school grads	0.166	0.397*	0.126	0.374*
	(0.190)	(0.203)	(0.188)	(0.199)
Distance to Budapest	−0.021	−0.169	−0.035	−0.198*
	(0.104)	(0.114)	(0.102)	(0.112)
Share of pop. inactive	−0.797***	−0.931***	−0.675***	−0.805***
	(0.229)	(0.229)	(0.232)	(0.229)
Unemployment Rate	0.239**	0.253**	0.247**	0.262**
	(0.118)	(0.115)	(0.116)	(0.113)
% population 60+	0.501***	0.546***	0.449***	0.491***
	(0.163)	(0.160)	(0.162)	(0.158)
Has University	0.351	0.198	0.449**	0.294
	(0.220)	(0.222)	(0.221)	(0.221)
Constant	1.245*	1.426**	1.036	1.206*
	(0.725)	(0.712)	(0.720)	(0.702)
Observations	169	169	169	169
Adjusted R^2	0.163	0.198	0.186	0.230
F Statistic	3.967***	4.460***	4.207***	4.859***

Table 3: Stepwise regressions. The effect and significance of the network features are preserved when including them only one at a time. *p<0.1; **p<0.05; ***p<0.01.

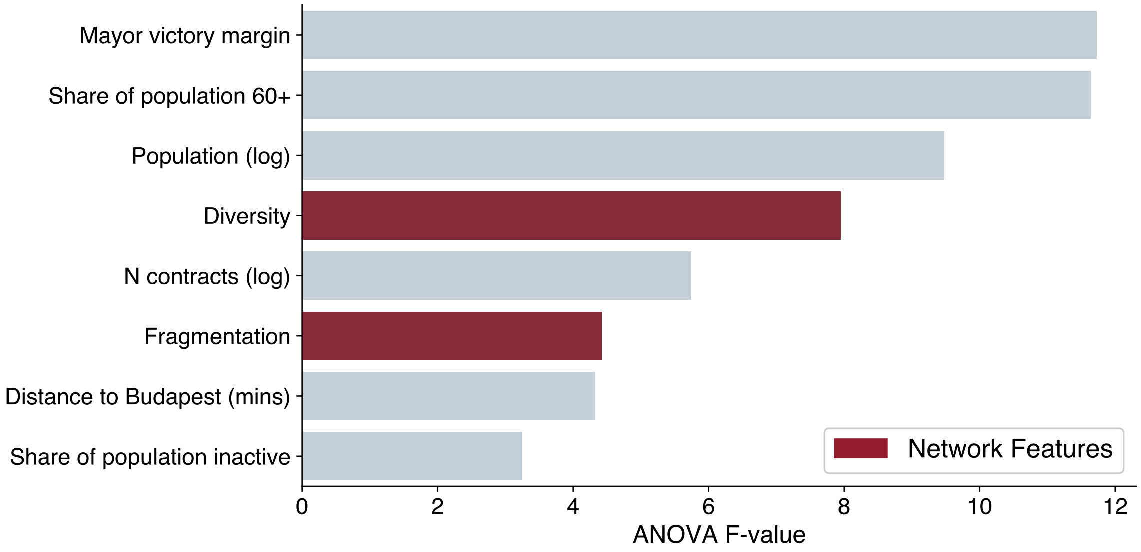

Figure 3: Analysis of Variance F-test feature importances of OLS regression predicting average settlement CRI. We only include significant features, and highlight the network-based social capital measures.

References

[1] R. Broms, C. Dahlström, and M. Fazekas. Political competition and public procurement outcomes. *Comparative Political Studies*, page 0010414019830723, 2019.

[2] Campante, Filipe R and Do, Quoc-Anh. Isolated capital cities, accountability, and corruption: Evidence from US states. *American Economic Review*, 104(8):2456–81, 2014.

[3] Q. Cao, M. Sirivianos, X. Yang, and T. Pregueiro. Aiding the detection of fake accounts in large scale social online services. In *Proceedings of the 9th USENIX conference on Networked Systems Design and Implementation*, pages 15–15. USENIX Association, 2012.

[4] M. Fazekas, I. J. Tóth, and L. P. King. An objective corruption risk index using public procurement data. *European Journal on Criminal Policy and Research*, 22(3):369–397, 2016.

[5] J. F. Hair, R. E. Anderson, R. L. Tatham, W. C. Black, et al. *Multivariate Analysis*. Prentice Hall, 1999.

[6] B. Lengyel, A. Varga, B. Ságvári, Á. Jakobi, and J. Kertész. Geographies of an online social network. *PloS one*, 10(9):e0137248, 2015.

[7] A. Mungiu-Pippidi. Controlling corruption through collective action. *Journal of Democracy*, 24 (1):101–115, 2013.

[8] U. Pfeil, R. Arjan, and P. Zaphiris. Age differences in online social networking–a study of user profiles and the social capital divide among teenagers and older users in myspace. *Computers in Human Behavior*, 25(3):643–654, 2009.

[9] B. Rothstein and E. M. Uslaner. All for all: Equality, corruption, and social trust. *World Politics*, 58(1):41–72, 2005.

Predictor	VIF
Fragmentation	1.407
Diversity	6.337
Income/capita	5.430
N contracts (log)	3.045
Population (log)	5.892
Rate iWiW use	2.885
Mayor victory margin	1.040
% high school grads	7.106
Share of pop. inactive	9.899
Unemployment Rate	2.360
Distance to Budapest	3.068
% population 60+	5.442
Has university	2.192

Table 4: VIF scores for model predictors.

[10] H.-E. Sung. Democracy and political corruption: A cross-national comparison. *Crime, Law and Social Change*, 41(2):179–193, 2004.

Dependent variable:	% Closed or single bid.		Average CRI	
	(1)	(2)	(3)	(4)
Fragmentation		0.143**		0.140**
(Bonding social capital)		(0.069)		(0.067)
Diversity		−0.358***		−0.440***
(Bridging social capital)		(0.138)		(0.134)
Income/capita	−0.324**	−0.351***	−0.323**	−0.356***
	(0.131)	(0.129)	(0.128)	(0.126)
N contracts (log)	−0.389***	−0.384***	−0.669***	−0.672***
	(0.118)	(0.118)	(0.116)	(0.115)
Population (log)	−0.064	0.036	0.176	0.318**
	(0.112)	(0.131)	(0.110)	(0.128)
Rate iWiW use	0.042	−0.001	0.105	0.052
	(0.094)	(0.094)	(0.092)	(0.092)
Mayor victory margin	0.176**	0.173**	0.174**	0.169**
	(0.070)	(0.069)	(0.069)	(0.067)
% high school grads	0.170	0.348**	−0.036	0.190
	(0.122)	(0.144)	(0.120)	(0.140)
Distance to Budapest	−0.089	−0.204**	0.048	−0.093
	(0.078)	(0.088)	(0.077)	(0.086)
Share of pop. inactive	−0.456***	−0.440***	−0.430***	−0.422***
	(0.138)	(0.138)	(0.135)	(0.134)
Unemployment Rate	0.058	0.064	−0.017	−0.011
	(0.079)	(0.078)	(0.078)	(0.076)
% population 60+	0.358***	0.329***	0.283***	0.251**
	(0.108)	(0.107)	(0.106)	(0.104)
Has University	0.289	0.289	0.406**	0.384*
	(0.204)	(0.208)	(0.200)	(0.202)
Constant	1.561***	1.540***	2.642***	2.652***
	(0.463)	(0.464)	(0.453)	(0.451)
Observations	305	305	305	305
Adjusted R^2	0.106	0.129	0.143	0.175
F Statistic	4.271***	4.452***	5.628***	5.974***

Table 5: Settlement-level regression results predicting two corruption risk indicators, including all towns issuing at least one contract a year on average from 2006 to 2014. Note that all features are standardized with mean 0 and standard deviation 1. Significance thresholds: *p<0.1; **p<0.05; ***p<0.01.
